# Prognostic tools at hospital arrival in acute myocardial infarction: copeptin and hepatocyte growth factor

**DOI:** 10.1186/s43044-022-00275-9

**Published:** 2022-04-28

**Authors:** María-Consuelo Pintado, Lara Maceda, María Trascasa, Ignacio Arribas, Raúl De Pablo

**Affiliations:** 1 Critical Care Unit, Hospital Universitario Príncipe de Asturias, Carretera Alcalá-Meco SN, 28805 Alcalá de Henares, Madrid Spain; 2 University of Alcalá, Alcalá de Henares, Madrid Spain; 3 Department of Biochemistry, Hospital Universitario Príncipe de Asturias, Carretera Alcalá-Meco SN, 28805 Alcalá de Henares, Madrid Spain; 4 Department of Biochemistry, Hospital Universitario Ramón y Cajal, Carretera de Colmenar Viejo Km 9.100, 28034 Madrid, Spain; 5 Critical Care Unit, Hospital Universitario Ramón y Cajal, Carretera de Colmenar Viejo Km 9.100, 28034 Madrid, Spain

**Keywords:** Hepatocyte growth factor, Copeptin, Myocardial infarction, Prognosis, Hospital mortality, Follow-up study

## Abstract

**Background:**

Prompt evaluation and treatment of acute coronary syndrome has demonstrated to reduce mortality. Although several biomarkers have been studied for risk stratification and prognostic purposes, none is recommended to guide treatment based on its prognostic value. Copeptin and hepatocyte growth factor have been associated with poor outcome in patients with acute myocardial infarction. The aim of this study is to evaluate the early prognostic value of measurements of copeptin and hepatocyte growth factor for hospital mortality risk and 1-year-follow-up mortality, in patients with acute myocardial infarction. In our retrospective observational study, we measured hepatocyte growth factor and copeptin in blood samples collected at hospital arrival in patients with acute myocardial infarction; and follow-up them until 1-year.

**Results:**

84 patients with were included in the study, mainly male (65%) with a median age of 70.3 ± 13.56 years. Hospital mortality was 11.9%. Plasma levels of copeptin at hospital arrival were statistically significant higher in patients who died during hospital admission (145.60 pmol/L [52.21–588.50] vs. 24.79 pmol/L [10.90–84.82], *p* 0.01). However, we found no statistically significant association between plasma levels of hepatocyte growth factor and hospital mortality (381.05 pg/ml [189.95–736.65] vs. 355.24 pg/ml [175.55–521.76], *p* 0.73). 1-year follow-up mortality was 21.4%. Plasma levels of copeptin at hospital arrival were higher in those patients who died in the following year (112.28 pmol/L [25.10–418.27] vs. 23.82 pmol/L [10.96–77.30], *p* 0.02). In the case of HGF, we also find no association between hepatocyte growth factor plasma levels and 1 -year follow-up mortality (350.00 pg/ml [175.05–555.08] vs. 345.53 pg/ml [183.68–561.15], *p* 0.68).

**Conclusions:**

In patients with acute myocardial infarction measurement of copeptin at hospital arrival could be a useful tool to assess the prognosis of these patients, since their elevation is associated with a higher hospital mortality and higher 1-year follow-up mortality. We have not found this association in the case of hepatocyte growth factor measurement.

## Background

Coronary heart disease is a leading cause of mortality in developed countries with a high incidence. Prompt evaluation and treatment has demonstrated to reduce mortality, especially in acute coronary syndrome (ACS), although it still remains elevated: 4–12% hospital mortality and around 10% 1-year mortality in ST-elevation myocardial infarction (MI), 6.3% 6-month mortality in non ST-elevation MI [1, 2].

Several biomarkers have been studied for risk stratification and prognostic purposes in acute coronary syndromes. The use of a single prognostic marker to guide treatment is currently not recommended [1, 2].

Copeptin is the C-terminal part of pro-arginine vasopressin. It is secreted in equimolar amounts than antidiuretic hormone in response to changes in plasma osmolarity and reduced cardiac output; although it exact function is unknown. Several studies had shown that it levels increases early on an acute MI (< 3 h), even when other biomarkers are still negative [3]. It is postulated that endogenous stress that occurs during MI activates the arginine vasopressin system independently of cardiac cell necrosis and leads to an increase of vasopressin copeptin secretion [4, 5]. A meta-analysis [6] of diagnostic accuracy of copeptin combined with cardiac troponin, to rule out acute MI in Emergency Department, showed a high sensitivity (96%) but low specificity (56%). So currently, it is not recommended for diagnoses of early acute MI [1]. In patients with acute MI, copeptin have been associated with poor outcome: higher mortality [7, 8], development of ventricular dysfunction [9] or heart failure [10].

Hepatocyte growth factor (HGF) is produced by stromal cells, being the liver the major organ through which HGF is eliminated from circulation. Originally identified as a mitogen of hepatocytes and, later, reported to have diverse activities in various cell types: regenerative, anti-apoptotic and anti-fibrotic effects [11]. In clinical studies, plasma HGF concentrations showed an early increase in patients with cardiac infarction (within 3 h after cardiac infarction), followed by a rapid decline in their levels during the first 24 h of MI, to remain stable for up to one year [12–14]. In other studies, HGF has been reported as a marker of arteriosclerosis [15], peripheral arterial occlusive disease [16] and incident coronary heart disease [17]; also, it has been associated with poor prognosis in acute coronary syndromes [12, 18].

The main objective of this study is to evaluate the early prognostic value of measurements of copeptin and HGF at hospital arrival for hospital mortality risk, in patients with acute myocardial infarction. The secondary objective is to explore the association of them with 1-year-follow-up mortality.

## Methods

### Study design and setting

This retrospective, observational study was carried-out in a University Hospital of Madrid, Spain, over a period of eight months. The study was approved by the Institutional Ethics and Clinical Trials Committee of University Hospital Príncipe de Asturias. Written informed consent was obtained from the patients. Neither, the researchers nor the subjects recruited in this study, received any fees or material incentives for their participation. This study did not receive financial or logistical support from any institution outside the Service or the Hospital where the study was carried out.

### Selection of participants

We included all consecutive patients diagnosed of type 1 acute MI at hospital arrival during an 8-months period. Patients younger than 18-years-old, at terminal stage of any illness, life expectancy of less than one year, or who refusal to sign the consent to participate in the study, were excluded from our study.

The final diagnosis was made by a Cardiologist, according to the clinical data, electrocardiogram, echocardiogram and the determination of cardiac troponin, following to the international clinical guidelines of acute coronary syndrome [1, 2, 19].

### Measurements and interventions

The data compiled from the patients included in our study were: demographic characteristics; risk factors for ACS; previous history of ACS; concomitant diseases; current treatment; Global Registry of Acute Coronary Events (GRACE) risk score [20] and Thrombolysis in Myocardial Infarction (TIMI) risk score [21]; in-hospital and after one-year follow-up mortality.

We retrospectively measured HGF and copeptin in blood samples collected from patients at hospital arrival, when the diagnosis of acute MI was confirmed by Cardiologist during the first 48 h after hospital admission. Prospectively, we follow-up the patients until hospital discharge and 1-year follow-up after twelve months of study inclusion. 1-year follow-up was carried out by telephone interview. If contact with participants was not possible, data were collected from the closest relatives, legal surrogate or medical records.

During the study period, plasma samples were obtained from all blood samples collected from patients at admission in Emergency Department for the study of myocardial enzymes. Plasma samples were obtained (heparin-litio BD Vacutanier®) after centrifugation of blood samples during seven minutes at 3500 revolutions per minute, divided into aliquots of 0.5 ml (ml), stored at − 40 °C and saved until their analysis. Only plasma samples of patients included in the study were analyzed. Samples from patients not included in the study were eliminated following the protocol of the biochemical laboratory, at the time the patient was excluded from the study.

HGF was measured in plasma samples by an enzyme-linked immunosorbent assay (Abcam®), at the time of evaluation; the lower limit of detection was 3 pg/ml (10% coefficient of variation).

Copeptin was measured in plasma simples by automated immunofluorescent assay for the quantitative determination (Brahms®); the lower limit of detection was 0.9 pmol/Liter (pmol/L) (20% coefficient of variation).

High-sensitivity Cardiac Troponin I (hs-cTnI) was measured in plasma samples with ADVIA Centaur XP® TnI-Ultra® assay (Siemens®), at the moment of evaluation and subsequent measurements at six-hour interval until maximum peak was reached. The minimum measurable concentration using this assay with a < 10% coefficient variation is 30 nanogram/Liter (ng/L). The samples for creatine-kinase measurement were collected at evaluation in the Emergency Department and processed in an ADVIA Chemistry 1800 analyser (Siemens®).

### Analysis

We valued if the measured variables followed a normal distribution by using the Kolmogorov–Smirnov test. Those quantitative variables that follow a normal distribution are expressed as means ± S.D. and compared using the Student’s t test; otherwise, those that do not follow a normal distribution variables are shown as medians and interquartile ranges and compared using the Mann–Whitney test. Qualitative variables are shown as percentages and compared by the chi-square test.

The level of statistical significance was set to a *p* ≤ 0.05, using bilateral contrasts, and results were expressed with 95% confidence intervals.

Logistic regression models were built for multivariate analysis to identify factors associated with hospital mortality. Univariate analysis of main variables registered was done. Predictor variables that were statistically significant when evaluated individually against hospital mortality were included in the forward stepwise multiple logistic regression analysis. The results are presented as odds ratio with the appropriate 95% confidence interval.

Statistical analysis was performed using SPSS 15.0 software (SPSS Inc. Chicago Illinois).

## Results

### Characteristics of study subjects

A total of 303 patients with suspected acute MI (acute chest pain and high levels of hs-cTnI at hospital arrival) were evaluated, but only 84 were finally diagnosed of acute MI and included in the study. The patients included were mostly male (65.5%) with a median age of 70.3 ± 13.56 years, being the most frequent risk factor for ACS the systemic arterial hypertension (75.0%), followed by smoking (59.5%) and dyslipemia (58.3%) (Table 1).

**Table 1:** Characteristics of study subjects

	All patients (n = 84)	Hospital mortality			1 year follow-up		
		Survivors (n = 74)	Non survivors (n = 10)	*p*	Survivors (n = 66)	Non survivors (n = 18)	*p*
Age, years	70.3 ± 13.6	68.4 ± 13.0	84.0 ± 9.0	0.001	67.6 ± 13.1	80.2 ± 10.7	< 0.001
Sex, male	55 (65.5%)	51 (68.9%)	4 (40.0%)	0.087	46 (69.7%)	9 (50.0%)	0.119
GRACE risk score	118.7 ± 34.8	114.2 ± 33.4	151.6 ± 27.4	0.001	109.6 ± 30.3	153.8 ± 28.9	< 0.001
STEMI	25 (29,8%)	22 (29,7%)	3 (30,0%)	1.000	20 (30,3%)	5 (27,8%)	0.835
Risk factors for ACS							
Systemic hypertension	63 (75.0%)	53 (71.6%)	10 (100.0%)	0.060	47 (71.2%)	16 (88.9%)	0.218
Diabetes mellitus	37 (44.0%)	33 (44.6%)	4 (40.0%)	1.000	26 (39.4%)	11 (61.1%)	0.100
Dislipemia	49 (58.3%)	44 (59.5%)	5 (50.0%)	0.570	38 (57.6%)	11 (61.1%)	0.787
Known family history of cardiovascular diseases	2 (2.4%)	2 (2.7%)	0 (0.0%)	1.000	2 (3.0%)	0 (0.0%)	1.000
Smoking	50 (59.5%)	45 (60.8%)	5 (50.0%)	0.520	42 (63.6%)	8 (44.4%)	0.141
Previous history of coronary artery disease	28 (33.3%)	24 (32.4%)	4 (40.0%)	0.720	17 (25.8%)	11 (61.1%)	0.010
Comorbidity:							
Hyperuricemia	3 (3.6%)	2 (2.7%)	1 (10.0%)	0.320	2 (3.0%)	1 (5.6%)	0.520
Kidney disease	12 (14.3%)	7 (9.5%)	5 (50.0%)	0.001	5 (7.6%)	7 (38.9%)	0.001
Lung disease	15 (17.9%)	13 (17.6%)	2 (20.0%)	1.000	10 (15.2%)	5 (27.8%)	0.210
Hepatic disease	1 (1.2%)	1 (1.4%)	0 (0.0%)	1.000	(1.5%)	0 (0.0%)	1.000
Cardiomyopathy	2 (2.4%)	1 (1.4%)	1 (10.0%)	0.220	0 (0.0%)	2 (11.1%)	0.040
Valvular heart	4 (4.8%)	3 (4.1%)	1 (10.0%)	0.400	1 (1.5%)	3 (16.7%)	0.027

The results are shown as number and percentage, except those marked with * that are expressed as median ± S.D

*ACS* Acute coronary syndrome, *STEMI* ST-segment elevation myocardial infarction

### Main results

#### Hospital mortality

Of the 84 patients included in the study, 10 (11.9%) died during hospital admission. The deceased patients were older (84.0 ± 9.0 years versus (vs.) 68.4 ± 13.0 years in survivors, *p* = 0.001), had a higher punctuation on GRACE risk score [20] (151.6 ± 27.4 vs. 114.2 ± 33.4 in survivors, *p* = 0.001) and more frequently had prior kidney disease (5 patients (50.0%) vs. 7 patients (9.5%), *p* = 0.001) (Table 1).

Plasma levels of copeptin at hospital arrival were statistically significant higher in patients who died during hospital admission (145.6 pmol/L [52.21–588.5] in non-survivors vs. 24.79 pmol/L [10.90–84.82], *p* 0.001). However, we found no statistically significant association between plasma levels of HGF and hospital mortality (Table 2).

**Table 2:** Outcomes

	Hospital mortality			1-year follow-up mortality		
	Survivors	Non-survivors	*p*	Survivors	Non-survivors	*p*
Copeptin (pmol/L)	24.79 (10.90–84.82)	145.60 (52.21–588.50)	0.01	23.82 (10.96–77.30)	112.28 (25.10–418.27)	0.02
HGF (pg/ml)	355.24 (175.55–521.76)	381.05 (189.95–736.65)	0.73	345.53 (183.68–561.15)	350.00 (175.05–555.08)	0.68
Hs-cTnI at inclusion (ng/ml)	0.70 (0.15–4.58)	2.71 (0.53–22.16)	0.10	0.72 (0.17–4.71)	2.11 (0.28–9.97)	0.61
Maximum peak Hs-cTnI (ng/ml)	11.71 (2.64–35.58)	9.85 (2.41–50.99)	0.90	13.80 (2.50–38.54)	5.15 (2.87–20.44)	0.28
Creatine kinase at inclusion (UI/l)	141.00 (108.00–429.50)	176.50 (108.5–259.00)	0.97	154.00 (114.00–443.50)	159.50 (88.25–255.25)	0.36

The results are shown as median (P25–P75)

*HGF* hepatocyte growth factor

In the multivariate analyses that we performed, we found that only age and copeptin were associated to hospital mortality (Table 3).

**Table 3:** Factors associated to hospital mortality

	Odds ratio (95% CI)	*p* value
*Univariate analysis*		
Age	1.120 (1.03–1.209)	0.004
Sex	0.295 (0.08–1.086)	0.066
HGF	1.000 (0.999–1.001)	0.713
Troponin at admission	1.030 (0.993–1.069)	0.114
Higher troponin	0.999 (0.989–1.010)	0.917
Copeptin	1.001 (1.001–1.007)	0.006
TIMI	0.887 (0.571–1.380)	0.596
GRACE	1.031 (1.009–1.054)	0.005
GRACE > 140	8.121 (1.980–33.317)	0.004
STEMI	1.176 (0.287–4.822)	0.822
Risk factors for ACS:		
Systemic hypertension	2.352E (0.000)	0.998
Diabetes mellitus	0.972 (0.277–1.380)	0.965
Dislipemia	0.750 (0.213–2.643)	0.654
Known family history of cardiovascular diseases	0.000 (0.00)	0.999
Smoking	0.716 (0.203–2.525)	0.603
Previous history of coronary artery disease	1.613 (0.456–5.706)	0.548
Comorbidity:		
Hyperuricemia	6.519 (0.959–44.292)	0.055
Kidney disease	7.593 (1.926–29.937)	0.004
Lung disease	1.521 (0.366–6.315)	0.564
Hepatic disease	0.000 (0.00)	1.000
Cardiomyopathy	9.000 (0.522–155.242)	0.130
Valvular heart	2.933 (0.278–30.936)	0.371
*Multivariate analysis*		
Age	1.138 (1.033–1.253)	0.009
Copeptin	1.005 (1.001–1.009)	0.011

*ACS* Acute coronary syndrome, *HGF* hepatocyte growth factor

#### Mortality at one-year follow-up

Follow-up at 1 year was possible in all patients included in the study. The global mortality at one-year follow-up was 21.43% (18 of the 84 patients included in the study). As occurs in hospital mortality, deceased patients during 1 year follow-up were older (80.2 ± 10.7 years vs. 67.6 ± 13.1 years in survivors, *p* > 0.001), had a higher punctuation on GRACE score [20] (153.8 ± 28.9 vs.109.6 ± 30.3 in survivors, *p* > 0.001) and more frequently had prior kidney disease ( (7 patients (38.9%) vs. 5 patients (7.6%), *p* = 0.001). They also had more frequent previous history of heart disease, either coronary artery disease (11 patients (61.1%) vs. 17 patients (25.8%), *p* = 0.010), cardiomyopathy (2 patients (11.1%) vs. 0 patients (0.0%), *p* = 0.040) or valvular heart disease (3 patients (16.7%) vs. 1 patient (1.5%), *p* = 0.027) (Table 1).

As in hospital mortality, we found that plasma levels of copeptin at hospital arrival were higher in those patients who died during the following year (112.28 pmol/L [25.10–418.27] in 1-year follow-up non-survivors vs. 23.82 pmol/L [10.96–77.30], *p* 0.02). In the case of HGF, we also find no association between HGF plasma levels and 1-year follow-up mortality (Table 2).

## Discussion

Our study has shown that, in unselected patients with acute MI, higher levels of copeptin at hospital arrival were associated to worse prognosis, as it is associated with higher hospital mortality and higher 1-year follow-up mortality. However, we did not find this association with HGF.

Copeptin have been associated with high mortality in patients with suspected acute coronary syndrome at hospital arrival in many previous studies. Balmelli et al. [22] found that high levels of copeptin measured at hospital arrival were associated with worse prognosis: higher hospital mortality and 1-year mortality, in their prospective study in patients presenting to the Emergency Department with symptoms suggestive of acute MI of less than 12 h; although, in this study, only 15.9% of patients were finally diagnoses of acute MI, being the other diagnoses unstable angina (14.0%), cardiac but noncoronary cause in 13.0%, noncardiac cause of chest pain in 48.4% and remained of unknown origin in 48.4% of patients included in the study. Maisel et al. [23] also found that high levels of copeptin were a powerful predictor of death at 180 days and hospitalization, in patients who arrived at hospital with chest pain within 6 h of pain onset; however, in this study the proportion of patients with acute MI was only 7.9%. Lattuca et al. [24] measured level of copeptin and cardiac troponin I at the beginning of percutaneous coronary intervention in unselected patients with acute ST-segment elevation myocardial infarction. They found that levels of copeptin were higher in patients who died during hospitalization, during the first 30 days after the myocardial infarction and at 1 year follow-up; performed a multivariate analysis associating one year mortality with the presence cardiogenic shock, increasing age, the presence of higher levels of copeptin (> 128.2 pmol/L) and radial access.

It has been demonstraed that the level of copeptin at admission has a superior prognostic compared to peak of cardiac troponin I. In this way, the ConTrACS study [25] evaluated the prognostic value of copeptin, measured at hospital arrival, in symptomatic patients with increased high sensitivity cardiac troponin T presenting to the Emergency Department. They demonstrated that patients with higher levels of copeptin had higher mortality in the entire cohort. Without differences when they analyzed only the group of patients who were finally diagnoses of acute coronary syndrome (27.6% vs 10.6% in patients with normal copeptin values, < 0.0001). Also, they found that the 30 days follow-up mortality were associated with higher levels of copeptin (4.5% vs 0.4% in patients with normal copeptin values, < 0.0001). Zellweger et al. [26] demonstrated that copeptin was strongly and independently associated with death during 720 days follow-up in diabetic patients with acute MI; even in those in which the diagnosis of acute MI was not finally confirmed.

One recent meta-analysis [27] evaluated the prognostic value of copeptin in patients with acute coronary syndrome and concluded that elevated copeptin was associated with higher mortality with a pooled sensitivity of 0.77% (95% CI: 0.59–0.89) and a pooled specificity of 0.60 (95% CI: 0.47–0.71). It included 6 studies, with a variable follow-up period between 30 days and 1 year.

Also, copeptin has been related with the development of post MI complications, as development of left ventricular dysfunction and heart failure after MI, what influences acute MI mortality [8, 9, 28].

In patients with ischemic heart disease, the prognosis value of serum levels of HGF has demonstrated contradictory results. In our study, we did not find an association between HGF levels and worse outcome in patients with acute MI.

Some authors have related high levels of HGF with a better prognosis. So, Yasuda et al. [29] measured HGF levels in 40 patients with acute MI who underwent coronary reperfusion therapy upon admission. They found an enhanced HGF secretion from the infarct region, which correlated inversely with the left ventricular end-diastolic volume index, left ventricular end-diastolic pressure and tau, and positively with left ventricular ejection fraction; so, they concluded that enhanced HGF secretion is associated with attenuation of ventricular remodeling and improvement in cardiac function. Heeschen et al. [30] found that high HGF serum levels in patients with refractory unstable angina were associated with low incidence of death and nonfatal myocardial infarction at six-month follow-up; although in this study, patients with higher HGF levels had more frequently collateral circulation and partial or complete retrograde filling in coronary angiogram study.

On the other hand, some studies show that HGF levels are significantly higher in patients with worse prognosis. Konopka et al. [12] measured levels of HGF as soon as possible after admission to hospital and 24 h afterward, in 104 patients with the first episode of ACS who were eligible for coronary angiography and, eventually, percutaneous coronary intervention. They found that 33 patients (32%) suffered the primary outcome of the study (a composite endpoint of death, MI, exacerbation of angina, heart failure, cardiovascular re-intervention, re-hospitalization due to cardiovascular causes and stroke). On them, the concentration of HGF observed was significantly higher than in patients without any complication, classified as the primary outcome during the three-month follow-up. This authors also measured HGF levels in 6 patients with acute MI during the first day of ACS (repeated 10 times during the first day) and once at discharge from hospital; and found a marked increase in HGF levels in the first measurement, that were reduced to almost normal values within 5 h, with a second increase of HGF levels during the first 24 h in those patients who had serious cardiovascular events in the acute stage of MI (cardiac arrest and early re-occlusion of the coronary artery) [31]. Lamblin et al. [14] found that levels of HGF were higher in patients who died or were rehospitalization for heart failure during 1-year follow-up after a first anterior wall Q‐wave MI. Susen et al.^18^ measured HGF levels at admission in 488 patients with acute MI referred for percutaneous coronary revascularization who were not receiving heparin and found that serum HGF levels were a strong and independent predictor of worse clinical events (a composite of death and non-fatal MI) during a median follow-up period of 14.9 months.

This study had several limitations. First, HGF and copeptin samples were obtained in a variable time since onset of symptoms, hence the rapid changes in serum levels may affect the results. Second, we did not make serial determinations of copeptin nor HGF, to see the evolution and peak maximum. Third, we cannot quantify exactly the clinical benefit associated with an improved risk stratification based on copeptin levels. Fourth, our study is only a descriptive study, not an interventional one, so we cannot evaluate the clinical impact of measurement of HGF and copeptin at hospital arrival.

Future studies are warranted to examine if those patients with high levels of copeptin could possibly benefit from revascularization of all the lesions found in the coronary angiography, prolong admission to the coronary unit or reinforcement of antiplatelet treatment, or even, anticoagulation therapy; closer follow-up or more rigorous control of treatment and risk factors. Fifth, we did not assess the relationship between the treatment received, HGF and copeptin levels and mortality.

We consider that the main strength of our study is that it has allowed us to obtain real world data, since it was carried-out under conditions of daily clinical practice.

## Conclusions

In conclusions, in patients with acute MI measurement of copeptin at hospital arrival could be a useful tool to assess the prognosis of these patients, since their elevation is associated with a higher hospital mortality and higher 1-year follow-up mortality. We have not found this association in the case of HGF measurement.

## Data Availability

The datasets generated and/or analyzed during the study are available from the corresponding author on reasonable request.

## Abbreviations

ACS: Acute coronary syndrome
GRACE: Global Registry of Acute Coronary Events
HGF: Hepatocyte growth factor
hs-cTnI: High-sensitivity Cardiac Troponin I
L: Liter
MI: Myocardial infarction.
ml: Milliliters
ng: Nanogram
pmol: Picomole
TIMI: Thrombolysis in myocardial infarction
vs.: Versus

## References

[1] Collet JP, Thiele H, Barbato E (2020). ESC Guidelines for the management of acute coronary syndromes in patients presenting without persistent ST-segment elevation. Eur Heart J.

[2] Ibanez B, James S, Agewall S (2017). 2017 ESC Guidelines for the management of acute myocardial infarction in patients presenting with ST-segment elevation. Rev Esp Cardiol (Engl Ed).

[3] Schurtz G, Lamblin N, Bauters C (2015). Copeptin in acute coronary syndromes and heart failure management: state of the art and future directions. Arch Cardiovasc Dis.

[4] Meune C, Zuily S, Wahbi K (2011). Combination of copeptin and high-sensitivity cardiac troponin T assay in unstable angina and non-ST-segment elevation myocardial infarction: a pilot study. Arch Cardiovasc Dis.

[5] Lippi G, Plebani M, Di Somma S (2012). Considerations for early acute myocardial infarction rule-out for emergency department chest pain patients: the case of copeptin. Clin Chem Lab Med.

[6] Raskovalova T, Twerenbold R, Collinson PO (2014). Diagnostic accuracy of combined cardiac troponin and copeptin assessment for early rule-out of myocardial infarction: a systematic review and meta-analysis. Eur Heart J Acute Cardiovasc Care.

[7] O'Malley RG, Bonaca MP, Scirica BM (2014). Prognostic performance of multiple biomarkers in patients with non-ST-segment elevation acute coronary syndrome: analysis from the MERLIN-TIMI 36 trial (Metabolic Efficiency With Ranolazine for Less Ischemia in Non-ST-Elevation Acute Coronary Syndromes-Thrombolysis In Myocardial Infarction 36). J Am Coll Cardiol.

[8] Khan SQ, Dhillon OS, O'Brien RJ (2007). C-terminal provasopressin (copeptin) as a novel and prognostic marker in acute myocardial infarction: Leicester Acute Myocardial Infarction Peptide (LAMP) study. Circulation.

[9] Kelly D, Squire IB, Khan SQ (2008). C-terminal provasopressin (copeptin) is associated with left ventricular dysfunction, remodeling, and clinical heart failure in survivors of myocardial infarction. J Card Fail.

[10] Voors AA, von Haehling S, Anker SD (2009). C-terminal provasopressin (copeptin) is a strong prognostic marker in patients with heart failure after an acute myocardial infarction: results from the OPTIMAAL study. Eur Heart J.

[11] Nakamura T, Mizuno S (2010). The discovery of hepatocyte growth factor (HGF) and its significance for cell biology, life sciences and clinical medicine. Proc Jpn Acad Ser B Phys Biol Sci.

[12] Konopka A, Janas J, Piotrowski W (2010). Hepatocyte growth factor–a new marker for prognosis in acute coronary syndrome. Growth Factors.

[13] Jimenez-Navarro MF, Gonzalez FJ, Caballero-Borrego J (2011). Coronary disease extension determines mobilization of endothelial progenitor cells and cytokines after a first myocardial infarction with ST elevation. Rev Esp Cardiol.

[14] Lamblin N, Bauters A, Fertin M (2011). Circulating levels of hepatocyte growth factor and left ventricular remodelling after acute myocardial infarction (from the REVE-2 study). Eur J Heart Fail.

[15] Nishimura M, Ushiyama M, Nanbu A (1997). Serum hepatocyte growth factor as a possible indicator of arteriosclerosis. J Hypertens.

[16] Yoshitomi Y, Kojima S, Umemoto T (1999). Serum hepatocyte growth factor in patients with peripheral arterial occlusive disease. J Clin Endocrinol Metab.

[17] Decker PA, Larson NB, Bell EJ (2019). Increased hepatocyte growth factor levels over 2 years are associated with coronary heart disease: the Multi-Ethnic Study of Atherosclerosis (MESA). Am Heart J.

[18] Susen S, Sautiere K, Mouquet F (2005). Serum hepatocyte growth factor levels predict long-term clinical outcome after percutaneous coronary revascularization. Eur Heart J.

[19] Thygesen K, Alpert JS, Jaffe AS (2018). Fourth Universal Definition of Myocardial Infarction. Circulation.

[20] Fox KA, Dabbous OH, Goldberg RJ (2006). Prediction of risk of death and myocardial infarction in the six months after presentation with acute coronary syndrome: prospective multinational observational study (GRACE). BMJ.

[21] Antman EM, Cohen M, Bernink PJ (2000). The TIMI risk score for unstable angina/non-ST elevation MI: a method for prognostication and therapeutic decision making. JAMA.

[22] Balmelli C, Meune C, Twerenbold R (2013). Comparison of the performances of cardiac troponins, including sensitive assays, and copeptin in the diagnostic of acute myocardial infarction and long-term prognosis between women and men. Am Heart J.

[23] Maisel A, Mueller C, Neath SX (2013). Copeptin helps in the early detection of patients with acute myocardial infarction: primary results of the CHOPIN trial (Copeptin Helps in the early detection Of Patients with acute myocardial INfarction). J Am Coll Cardiol.

[24] Lattuca B, Sy V, Nguyen LS (2019). Copeptin as a prognostic biomarker in acute myocardial infarction. Int J Cardiol.

[25] Waldsperger H, Biener M, Stoyanov KM (2020). Prognostic value of elevated copeptin and high-sensitivity cardiac troponin t in patients with and without acute coronary syndrome: the ConTrACS Study. J Clin Med.

[26] Zellweger C, Wildi K, Twerenbold R (2015). Use of copeptin and high-sensitive cardiac troponin T for diagnosis and prognosis in patients with diabetes mellitus and suspected acute myocardial infarction. Int J Cardiol.

[27] Lu J, Wang S, He G (2020). Prognostic value of copeptin in patients with acute coronary syndrome: a systematic review and meta-analysis. PLoS ONE.

[28] Xu L, Liu X, Wu S (2018). The clinical application value of the plasma copeptin level in the assessment of heart failure with reduced left ventricular ejection fraction: a cross-sectional study. Medicine (Baltimore).

[29] Yasuda S, Goto Y, Baba T (2000). Enhanced secretion of cardiac hepatocyte growth factor from an infarct region is associated with less severe ventricular enlargement and improved cardiac function. J Am Coll Cardiol.

[30] Heeschen C, Dimmeler S, Hamm CW (2003). Prognostic significance of angiogenic growth factor serum levels in patients with acute coronary syndromes. Circulation.

[31] Konopka A, Janas J, Stepinska J (2012). Hepatocyte growth factor concentration during the first day of acute coronary syndrome. Arch Med Sci.

